# The Rudolph Leuckart Celebration

**Published:** 1895-11

**Authors:** Chas. Wardell Stiles

**Affiliations:** U. S. Department of Agriculture, Washington, D. C.


### THE RUDOLPH LEUCKART CELEBRATION.

EDITOR OF THE JOURNAL OF COMPARATIVE MEDICINE, ETC.:

Several months ago the following circular was sent out from Leipzig, signed by about a hundred and fifty scientists from the various parts of the world:

"*Zur Feier des am* 13 *December*, 1895, *stattfindenden fünfzigjährigen Doctorjubiläums von Rudolf Leuckart*, dem Nestor unter den deutschen Zoölogen, dessen Wirken weit über den Kreis seiner Specialwissenschaft hinausreicht, fordern die ergebenst Unterzeichneten zu Beiträgen auf. Im Herzen seiner zahlreichen Verehrer steht es fest, dass der seltene Tag nicht vorbei gehen darf ohne ein dauerndes Zeichen der Erinnerung. Wir gedenken von einem hervorragenden Bildhauer 'Leuckart's Marmorbüste' herstellen zu lassen und sie zugleich mit einer künstlerisch ausgestatteten Adresse zu überreichen.

"Wir wenden uns an alle, welche in ihrem geistigen Entwickelungsgange sein Wirken und seinen Einfluss verspürt haben, dass sie zu einer würdigen Ehrung des Jubilars beisteuern.

"Da es unmöglich ist, die Adressen aller seiner Schüler, namentlich derer, die nicht Zoologen von Fach geblieben sind, zu erlangen, so bitten wir diejenigen Herren, welche der allgemeinen Anregungen, die sie aus Leuckart's Vorlesungen in ihren Beruf mit hinausgenommen haben, in Dankbarkeit gedenken, dass sie in ihren Kreisen durch Verbreitung dieses Aufrufs in unserem Sinne thätig sind.

"Beträge werden erbeten an Herrn Carl Graubner (C. F. Winter's Verlag, Leipzig, Johannesgasse 8), welcher das Amt des Schatzmeisters freundlichst übernommen hat."

Within a few weeks of the receipt of the circular by American zoölogists I received a number of inquiries from various sources asking for further information regarding the subject, but was unable to reply to these inquiries, as I had not learned the detailed plans of the Leipzig Committee. At present, however, I can furnish some of the desired information.

It is the intention of the Leipzig Committee to have a life-size marble bust of the Geheimrath made and to present it to him on December 13th, and it is understood that the bust will eventually be deposited in the University of Leipzig or in the Leipzig Gallery. The statue will be made by one of the most prominent sculptors of Germany, who attended Leuckart's lectures this last semester, unbeknown to the lecturer, in order to study his expression. The estimated cost is 4000 marks, of which about 1000 marks had been subscribed before September 1st. Should more money be collected than is necessary it will probably be spent for photographs of the bust, which will be sent to persons who have forwarded subscriptions.

The subscriptions thus far made vary from 10 to 200 marks, most of them being in sums of 20 to 50 marks.

It is not intended to confine the subscriptions to Leuckart's pupils, for a number of other persons have expressed their desire to contribute. The Leipzig Committee therefore extends a cordial invitation to all admirers of the Geheimrath to join in the celebration, and I would therefore urge all of Leuckart's pupils in this country to bring this circular to the attention of their scientific and medical friends.

Subscriptions can be sent to Carl Graubner, as announced in the original circular, or to me. At the request of Dr. Simroth, the moving spirit in the undertaking, I have agreed to receive American subscriptions and forward the same in one sum to Leipzig.

Ch. Wardell Stiles.

U. S. Department of Agriculture,
Washington, D. C.

